# Psychological Distress Among Health Care Workers in Health Facilities of Mettu Town During COVID-19 Outbreak, South West Ethiopia, 2020

**DOI:** 10.3389/fpsyt.2021.574671

**Published:** 2021-06-23

**Authors:** Mohammedamin Hajure, Bekem Dibaba, Shuayib Shemsu, Defaru Desalegn, Mohammed Reshad, Mustefa Mohammedhussein

**Affiliations:** ^1^Department of Psychiatry, College of Health Sciences, Mettu University, Mettu, Ethiopia; ^2^Department of Midwifery, College of Health Sciences, Mettu University, Mettu, Ethiopia; ^3^Department of Public Health, College of Health Sciences, Mettu University, Mettu, Ethiopia

**Keywords:** coronavirus, health care provider, Mettu, Ethiopia, 2020

## Abstract

**Background:** During any of the infectious disease outbreak, health care workers were at increased risk of being infected, and psychological distress was a common phenomenon. Therefore, the study aimed to assess the psychological distress related to COVID-19 among healthcare workers in Mettu town.

**Methods:** A cross sectional study was conducted from May 1–15, 2020 using convenient sampling techniques among 127 health care providers during COVID-19 pandemic in the Mettu town. Self-administered questionnaire was used to collect information. Depression and anxiety were evaluated as subscales from the Depression Anxiety Stress Scale (DASS-21). Psychological distress related to COVID-19 was measured using the Impact of Event Scale Revised (IES-R). Data analysis were done using SPSS version 24. Chi-square test was used to find the association between the outcome and demographic variables. Multivariable logistic regression analyses were used to evaluate the significance of the association at *P*-value < 0.05.

**Result:** Using IES-R scale, 40.2% of the participants reported to have the symptoms of psychological distress. The majority of the participants reported mild psychological distress (37%) followed by moderate psychological distress (29%). The multivariate logistic regression analysis revealed that the odds of psychological distress were found to be higher among health care providers who reported to have depressive symptoms, and those who used alcohol, khat and tobacco in the past 3 months shows a significant association with psychological distress.

**Conclusion:** Our findings revealed that the COVID-19 pandemic had exerted major psychological distress on health care providers. So the findings, seek attention for early psychological intervention needed to manage psychological distress in health care providers regarding identified factors.

## Introduction

Healthcare workers are at the front line of any outbreak and are more likely to be exposed to hazards that put them at risk of infection which includes exposure to pathogen, extended working hours, psychological grief, fatigue, occupational burnout, stigma, and physical violence are among the hazards that healthcare workers come across during such an outbreak (1).

Research has shown that frontline health care workers who involved in direct patient care experience anxiety at greater levels and have poorer outcomes. Frontline staffs working in high-risk atmosphere are more likely to have fear of being infected and infecting others as well as experience higher levels of occupational stress, fatigue, and burnout (2). These in turn can serve as predecessors to more serious conditions which comprises anxiety, depression, substance use, and symptoms of post-traumatic stress disorder (3).

Frontline healthcare workers (HCWs) were making a sacrifice to fight the COVID-19 pandemic, while experiencing an increased work overload and the risk of contracting infection. similar to the outbreak of severe acute respiratory syndrome (SARS) in Hong Kong in 2003, some patients and health professionals would be traumatized by the COVID-2019 outbreak and suffer from persistent psychiatric manifestations even after the outbreak (4).

Health care worker's reaction to the stressor with the same set of circumstances may vary and the level of their job stress determined by the subjective overload related to the views of their condition and the coping strategies (5). Healthcare responders are at higher risk for traumatic stress reactions because their work repeatedly exposes them to highly stressful situations (6). A group at a particularly high risk is represented by physicians, and nurses working in emergency units and resuscitation departments (7). During the outbreak of SARS in 2003 in Toronto, about 27% of the health care providers reported emotional distress (8). Study in the western tertiary care centre, Germany showed that well-trained and dedicated healthcare workers can cope well with the stress of caring for a severely ill Ebola patient (9). During the 2003 SARS-CoV outbreak in Taiwan, (27% of health care workers) in the emergency department and in the psychiatric ward developed post-traumatic stress disorder (PTSD) (10).

A cross-sectional survey conducted among 338 Israeli dentists and dental hygienists found the prevalence of psychological distress 11.5%, results from this study revealed the elevated psychological distress among those who have background illness, fear of contracting COVID-19 from the patient, and a higher subjective overload (11). During the 2015 Korean MERS- COV outbreak, stigma and hardiness were found to have had a direct impact on the psychology of health personnel working in public hospitals (12). the finding from Hubei province, Wuhan in China among healthcare workers exposed to 2019 Coronavirus disease (COVID-19) found that the prevalence of psychological distress was 71.2% (13). In other ways, the magnitude of the psychological distress among healthcare workers during the COVID-19 pandemic in Ethiopia ranges from 42% (14) in Dessie city to 78.3% (15) in Jimma town.

The psychological consequences of the COVID-19 pandemic were particularly serious for healthcare providers because of higher levels of exposure. The mental health effects arising among health care providers may exert its impact on quality of health care provision or treatment (11). Due attention should be given to health care workers who have been risking themselves to fight the battle against COVID-19. Confinement, separation from loved ones, and reduced social and physical contact is a major cause of boredom, frustration, and distress for HCWs. concern about their own health condition or that of family members, Loss of income and financial strain concern about treatment requirements may be a significant stressor for healthcare workers (16).

COVID-19 has infected over 570,000 health workers and from these about 2,500 was killed in United States of Americas while making a sacrifice to save the lives of others (17). As of 25 April, Ethiopia counts 252,279 COVID-19 cases, including 59,979 active cases and 3,551 deaths (1.4 per cent case fatality rate) (18). These will cause an additional burden of stress to health care providers working in other parts of the country.

Currently there are only couple of studies concerning the psychological distress of COVID-19 and its correlates among health care providers in Ethiopia. So this study targeted to assess the psychological impacts of COVID-19 and its correlates among health care providers in Mettu town, south western Ethiopia.

## Methods

### Study Design, Period, and Area

A cross-sectional study was conducted. The study was conducted from May 1 to 15, 2020 in health facilities of Mettu town, the zone city, situated in the south-western region of Ethiopia, 600 km away from the capital, Addis Ababa. There were one referral hospital, one health centre, six private clinics and 11 private pharmacies in the town.

### Study Population

All healthcare workers from public and private health facilities who are available during the data collection period were included in the sample. Acutely ill workers who were unable to fill the questionnaires were excluded. In these sense, all the available healthcare providers meeting the eligibility criteria and working in the town were included in the study so as increase the sample size as a response to the emergency condition (COVID-19 pandemic). Convenience samples of 127 healthcare workers in the Mettu town were participated in the study.

### Data Collection Procedure and Tools

Self-administered structured questionnaires were used to collect information. Questionnaires to assess the demographic, substance use and stress related factors were developed after extensive review of similar or related articles carried out. We have used instruments which were validated in various contexts, even though not all instruments were validated in Ethiopia. All screening instruments were translated to the local language Afaan Oromo and were also pretested. Data were obtained from respondents working in (public hospital, health centre, and private health facilities) found in the city. All data collector tried to keep safety of the respondents and themselves by wearing masks, using hand sanitizers and also practicing social distancing even though it is self-administered. Variables such as feeling of healthcare workers to resign, thought of accepting the risk of exposure and believe to recover after infected with the virus were considered after searching a literatures.

Catastrophic psychological trauma caused by unexpected events was measured by the Impact of Event Scale (IES-R). This tool was first developed by Horowitz in 1979 (19). It was found to have good internal consistency in previous studies (20), and has cronbach α of 0.92 in the present study. This tool contains 5-point likert type scale ranged from 0 = never to 4 = always and measures in three dimensions: intrusion symptoms, avoidance symptoms, and high arousal symptoms. The item can also have classification with score range of IES-R scale as, 0–8 as subclinical, 9–25 as mild, 26–43 as moderate and 44–88 as severe. In this study scoring over 33 was considered as a cut off for a “probable PTSD case or psychological distress” (21).

Depression and anxiety were assessed by the depression and anxiety subscales from the DASS-21. DASS-21 is a validated and reliable instrument capable of differentiating symptoms of depression, anxiety, and stress (22). Scores obtained on these subscales were dichotomized. Accordingly, in the current study we have targeted only depression and anxiety as an explanatory variable to see its association with psychological distress. In these context, the average binary score of DASS-21 for the current study was 10 for depression subscale, eight for anxiety subscale. Those falling in moderately, severely and extremely severely depressed and anxious categories were considered as depressed and anxious, respectively.

In other ways, sum scores of 0–9 for depression and 0–7 for anxiety were considered as normal. Sum scores of 14–20 for depression and 10–14 for anxiety were considered as moderate. Finally, sum scores of 21–27 for depression and 15–19 for anxiety were considered as severe. Any scores above these were considered as extremely severe. As explained above it is quantitative measures (23). The cronbach's α measured in current study was 0.963 for depression, and 0.971 for anxiety which shows good internal consistency for the scale.

**Psychological distress:** Scoring over 33 was considered as a cut off points using the Impact of Event Scale revised (IES-R). The item can also be categorized into levels, the score range of IES-R scale was 0–8 as subclinical, 9–25 as mild, 26–43 as moderate, and 44–88 as severe (21).

**Depression and anxiety:** It was measured by the depression and anxiety subscale of Depression, Anxiety and Stress Scale (DASS-21). Score ≥10 and ≥8 was used to define depression and anxiety, respectively (23).

**Current substance use:** Use of alcohol, tobacco and khat once or more in the past 3 months.

**Health care workers:** In the current refers to personnel licensed in any of health fields.

**Medical health care workers:** In the current study refers to health personnel licensed in medical doctors, laboratory technician, or expert and pharmacy.

**Non-medical health care workers:** In the current study refers to health personnel licensed in any field of health other than medical doctors, laboratory technicians or experts and pharmacists.

### Ethical Clearance

The study was carried out after ethical clearance was obtained from the ethical review board of Mettu University faculty of health and medical sciences. Permission letter was obtained from the department of psychiatry and written informed consent was obtained from each study participant the study was carried out in accordance to the principles embodied in the Declaration of Helsinki. Health care providers were informed about the aims of the study, the right to participate or refuse to participate in the study, and Confidentiality of information was ensured.

### Statistical Analysis

Once all necessary data was obtained, and checked for completeness. Data were coded, entered into Epi-Data version 3.1 and were analyzed using SPSS version 20.0. Chi-square was used to find the association between the outcome and independent variables. Multivariable logistic regression analysis was used to evaluate the significance of the relationship between dependent and independent variables at *p*-values of <0.05.

## Results

### Socio-Demographic Characteristics of the Study Participants

A total of 127 healthcare workers was enrolled in the study. Eighty-six (67.7%) were males. The mean age of the respondent was 31.89 (SD = 5.95) years. Most of them 74 (58.3%) were married. Regarding the educational status majority 77 (60.6%) were first degree and above holders. Concerning the substance use characteristics, khat (amphetamine like psychoactive substance) use in the past 3 months was reported in about 57 (45%) of the respondents, and about one third reported current tobacco smoke. More than half of the study participants, 76 (59.8%) and 68 (53.5%) were non-medical healthcare workers and lived within a household member of three or below. Majority believed not to recover if infected with the virus, 104 (81.9%) (Table 1).

**Table 1:** Sociodemographic, substance use and other clinical characteristics of study participants (*N* = 127).

**Variables**	**Category**	**Frequency (*N*)**	**Percent (%)**
Sex	Male	86	67.7
	Female	41	32.3
Age in years	≤31	71	55.9
	>31	55	43.3
Work experience	≤3	101	79.5
(in years)	>3	26	20.5
Marital status	Never married	53	41.7
	Married	74	58.3
Religion	Muslim	32	25.2
	Orthodox	44	34.6
	Protestant	51	40.2
Educational status	Diploma	50	39.4
	1st degree and above	77	60.6
Occupation	Medical HCWs	51	40.2
	Non-medical HCWs	76	59.8
Household family size	≤3	68	53.5
	>3	59	46.5
Current khat use	No	70	55.1
	Yes	57	44.9
Current alcohol use	No	76	59.8
	Yes	51	40.2
Current tobacco use	No	81	63.8
	Yes	46	36.2
Health workers felt to resign	No	116	91.3
	Yes	11	8.7
Thought of accepting risk of exposure	No	36	28.3
	Yes	91	71.7
Believe to recover if infected with the virus	No	104	81.9
	Yes	23	18.1

*“Khat, or qat, is a psychoactive stimulant substance (in the form of shrub) that has been chewed for centuries by people from the Horn of Africa and Arabian peninsula.”*

*HCW, health care workers*.

### Prevalence of Psychological Distress Among Healthcare Workers

**Figure 1:**
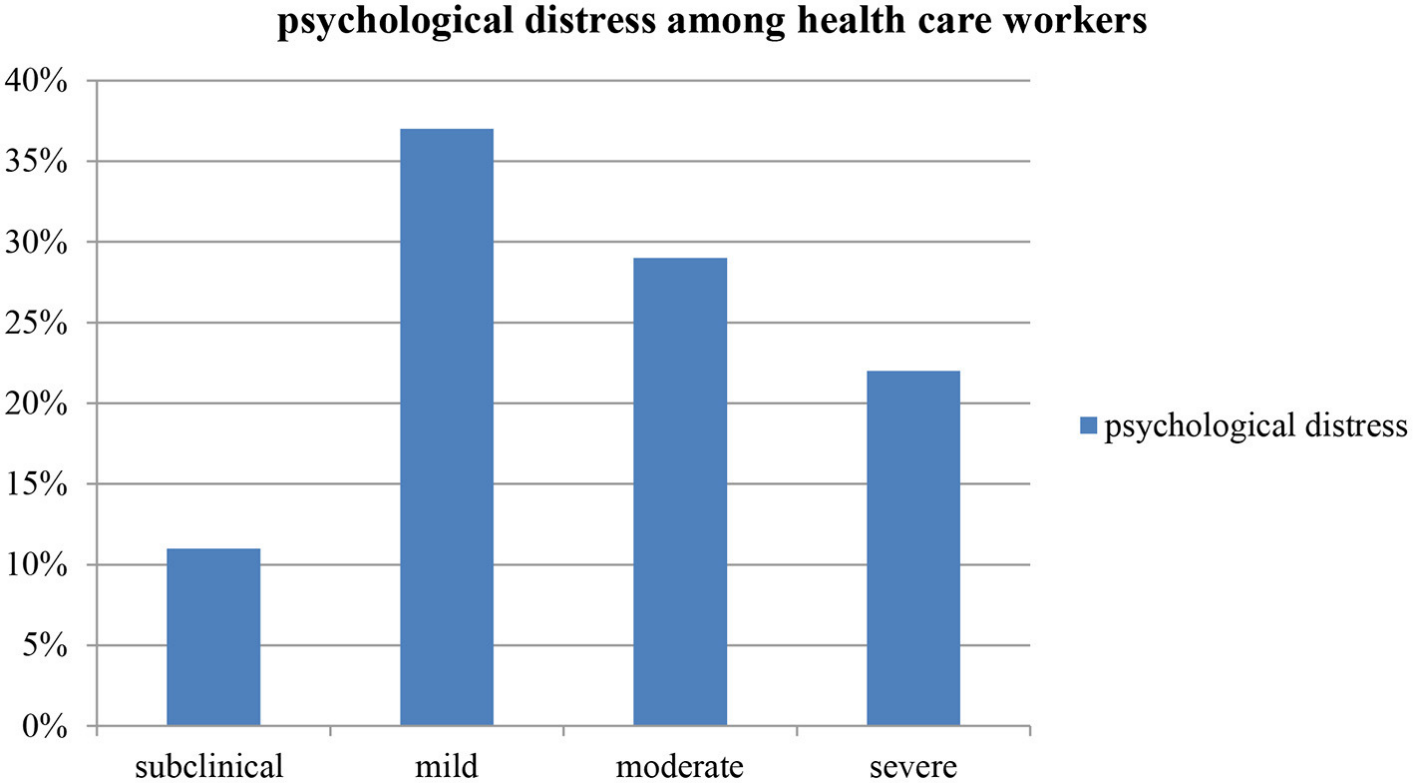
Level of psychological distress among HCWs in Mettu town, Ethiopia, 2020.

The prevalence of psychological distress among HCWs was 40.2% (95% CI 31.5, 48.0). Of all participants, 11% reported subclinical psychological impact, 37% rated mild psychological distress, and 29 and 22% of them reported moderate and severe psychological distress, respectively (Figure 1). Nearly about half of the health care workers showed positive for psychological distress (score of >33). On the other hand, considering the symptom cluster of IES-R scale, Avoidance symptoms were found to be the most concerned among health care workers compared to hyper arousal and intrusion symptom subscales (Table 2).

**Table 2:** Descriptive analysis of IES-R subscale among health care workers (*N* = 127).

**Variable**	**Mean**	**SD**	**Minimum**	**Maximum**
Avoidance symptoms	11.1	6.19	0	29
Hyper arousal symptoms	7.48	4.83	0	18
Intrusion symptoms	9.83	6.09	0	22

### Factors Associated With Psychological Distress Among Health Care Workers

In our study more male 33 (38.4%), and married 29 (39.2%) participants were found to have lower psychological distress compared to females (*P*-value = 0.552) and those who never married participants (*P*-value = 0.446), respectively. Participants with first degree and above, 33 (42.9%) (*P* = 0.441), and with family size of more than three 31 (39.2%) (*P* = 0.787), had higher psychological distress compared to their counterparts (Table 3). As shown in Tables 3, 4, participants fitting to psychological distress were more likely to be medical health workers (*P* = 0.043), those with depression (*P* < 0.001), and current alcohol (*P* < 0.001), current tobacco (*P* < 0.001), and current khat use (*P* < 0.001).

**Table 3:** Comparing sociodemographic characteristics with psychological distress (*N* = 127).

**Variables**	**Category**	**Psychological stress**		**Chi-square**	*p*-value^*^
		**Yes**	**No**		
Sex	Male	33 (38.4%)	53 (61.6%)	0.053	0.552
	Female	18 (43.9%)	23 (56.1%)		
Age in years	≤31	31 (43.1%)	41 (56.9%)	0.068	0.446
	>31	20 (36.4%)	35 (63.6%)		
Work experience (in years)	≤3	43 (42.6%)	58 (57.4%)	0.097	0.274
	>3	8 (30.8%)	18 (69.2%)		
Marital status	Never married	22 (41.5%)	31 (58.5%)	0.023	0.793
	Married	29 (39.2%)	45 (60.8%)		
Educational status	Diploma	18 (36.0%)	32 (64.0%)	0.068	0.441
	1st degree and above	33 (42.9%)	44 (57.1%)		
Occupation	Medical HCWs	15 (29.4%)	36 (70.6%)	0.180	0.043
	Non-medical HCWs	36 (47.4%)	40 (52.6%)		
Household family size	≤3	31 (39.2%)	48 (60.8%)	0.024	0.787
	>3	20 (41.7%)	28 (58.3%)		

*HCW, health care workers*.

* *Chi-square*.

**Table 4:** Comparing substance use and other clinical characteristic with psychological distress (*N* = 127).

**Variables**	**Category**	**Psychological stress**		**Chi-square**	*p*-value^*^
		**Yes**	**No**		
Current khat use	No	14 (20.0%)	56 (80.0%)	0.456	*P* < 0.001
	Yes	37 (64.9%)	20 (35.1%)		
Current alcohol use	No	13 (17.1%)	63 (82.9%)	0.574	*P* < 0.001
	Yes	38 (74.5%)	13 (25.5%)		
Current tobacco use	No	17 (21.0%)	64 (79.0%)	0.519	*P* < 0.001
	Yes	34 (73.9%)	12 (26.1%)		
Depression	No	19 (26.4%)	53 (73.6%)	0.321	*P* < 0.001
	Yes	32 (58.2%)	23 (41.8%)		
Anxiety	No	22 (35.5%)	40 (64.5%)	0.093	0.294
	Yes	29 (44.6%)	36 (55.4%)		
HCW felt to resign	No	44 (37.9%)	72 (62.1%)	0.147	0.096
	Yes	7 (63.6%)	4 (36.4%)		
Thought of accepting risk of exposure	No	16 (44.4%)	20 (55.6%)	0.055	0.535
	Yes	35 (38.5%)	56 (61.5%)		
Believe to recover if infected with the virus	No	42 (40.4%)	62 (59.6%)	0.010	0.912
	Yes	9 (39.1%)	14 (60.9%)		

*HCW, health care workers*.

* *Chi-square*.

However, the multivariate logistic regression analysis showed that the odds of psychological distress were higher among those who reported to have depression [AOR = 10.54; 95% CI (2.87, 38.7)], current tobacco use [AOR = 6.76; 95% CI (2.15, 21.2)], current use of khat [AOR = 5.74; 95% CI (1.83, 18.1)], current alcohol use [AOR = 6.28; 95% CI (2.03, 19.5)], these factors were independently associated with psychological distress. Household family size, work experience and presence of anxiety symptoms were found to have no association with psychological distress (Table 5).

**Table 5:** Multivariable logistic regression examining the associations between psychological distress and associated factors among HCW [*N* = 127].

**Study variable**	**Psychological distress**		**OR** **[95% CI adjusted]**	***P*-value^α^**
	**Yes *n* [%]**	**No *n* [%]**		
Depression				
Yes	32 (58.2%)	23 (41.8%)	10.5 [2.87–38.7]	<0.001^**^
No	19 (26.4%)	53 (73.6%)	1.00	
Khat use				
No	14 (20.0%)	56 (80.0%)	1.00	0.003^*^
Yes	37 (64.9%)	20 (35.1%)	5.74 [1.83–18.1]	
Tobacco use				
No	17 (21.0%)	64 (79.0%)	1.00	0.001^*^
Yes	34 (73.9%)	12 (26.1%)	6.76 [2.15–21.2]	
Alcohol use				
No	13 (17.1%)	63 (82.9%)	1.00	0.001^*^
Yes	38 (74.5%)	13 (25.5%)	6.28 [2.03–19.5]	

*OR, Odds ratio; CI, Confidence interval*.

** *p < 0.001;*

* *p < 0.01; 1.0 = Reference group*.

α *Adjusted for control variables in the table*.

## Discussion

This cross-sectional study included 127 participants, finding showed greater proportion of healthcare workers reported to have psychological distress during COVID-19 pandemic in Mettu town, southwest Ethiopia. Our findings present concerns about the psychological well-being of health care provider during the COVID-19 pandemic. In this study, the IES-R instrument was dichotomized based on the standardized cut off points and the probable psychological distress was reported to be 40.2%. Furthermore, three subscales were calculated (Intrusion, hyper arousal and avoidance) providing an indication of the level of distress experienced from low to high score. Accordingly, subclinical (11.0%), mild (37.0%), moderate (29.9%) and severe (22.0%) of psychological distress were reported among study participants.

Prevalence of psychological distress among health care workers in this study was higher than the study in Taiwan 11% (24) and lower than the result of study from Korea 51.5% (25), Dessie 42% (13) and Jimma 78.5% (26). A possible reason for the discrepancy might account to instruments used [(Kasseler-10 (13)) vs. current study (IES-R)], cut off points used, sample size [larger sample in Korean study (1,800 participants) (25) vs. current study (127 participants)], period of study and a sampling method [systematic sampling method vs. current study (convenience sample)].

On the other hand, the study done in Wuhan, China among health care providers showed a higher prevalence of psychological distress 71.2% than the current study (13). Healthcare workers in Wuhan, China the origin and the epicenter of the Covid-19 pandemic were the first exposed group to the virus and responded with significant levels of distress supported by the finding of study showed healthcare workers outside Hubei province was associated with lower risk of experiencing distress (13).

The current study indicated that health care providers who reported to have depression, and who reported to have used alcohol, tobacco and khat in the past 3 months were more likely to experience psychological distress. Our study also found increased odds of distress among respondents with underlying depression, providing a clue to target health care providers with depression to help them in managing their stress. The interconnection of depression and psychological stress is explained interms instability in immune function (26), the role of hypothalamic-pituitary-adrenal (HPA) axis and sympathetic nervous system (27).

In this sense, it was shown that COVID-19 pandemic is extremely affecting mental health status of the individuals extensively, generating symptoms of psychological distress (28). In the current study, the odds of having psychological distress among respondents who have used alcohol in the past 3 months were higher compared to non-user. This may be due to the effect of drinking which has the potential to threaten both our mental and physical health. This was supported by previous study showed the association between traumatic life events and risky drinking (29) which prompts risk of developing psychological distress.

Our study revealed that smoking tobacco in the past 3 months is associated with increased odds of developing psychological distress. This may be explained from effect of cigarettes to be harmful and increased the risk of heart disease and lung disorders. So that the COVID-19 virus affects the respiratory tract subsequently increasing the risk of developing psychological distress.

Study showed an increased risk of psychological distress thought to influence the pathogenesis of physical diseases by causing negative affective states (e.g., feelings of anxiety and depression), which in turn exert direct effects on behavioral patterns or biological processes on that effect disease risk as in COVID-19 infection (30). So an increased distress among those patients might be explained interms of its possible relation with risky behavior such as smoking (31). In contrast to this, smoker can also underestimate the detrimental effects of smoking on their health (32).

Individuals who had chewed khat in the past 3 months were 5.74 times more likely to have psychological distress when compared to their counterparts. This could be from the norms of the local community to chew khat (amphetamine like stimulant substance) in the forms of social gatherings which are against the COVID-19 prevention measures. So this might have contributed to increased fear states to contract the disease.

Furthermore, the present study showed that over four-fifths of respondents believed that they may not recover if infected with COVID-19. Even though we could not ascertain about the contributing factors toward this believe, we thought that issues such as lack of resources, lack of access to up to date health information and misinformation regarding the infection could account toward the contributing factors. In view of this, in particular misinformation, previously conducted studies have shown that, misinformation was critical issues in the face of the current pandemic and may cause or exacerbate psychological stress among the general public, and may in the future intensify anxiety and other significant stress disorder particularly in the occasion of a new wave of infections (33, 34).

### Limitation of the Study

The study has several limitations. Considering the limited availability of resources and urgent or alarming effect of the COVID-19 pandemic outbreak, we implemented the convenience sampling technique. This sampling strategy was not based on a random selection of the sample, and the study population did not reveal the actual pattern of the general population. In other ways authors have not included significant variables like clinical history of mood and/or anxiety disorder which might affect the outcome under study. Prospective studies addressing large sample will be essential to recognize the psychological impacts of COVID- 19 pandemic outbreak on health care providers.

### Conclusion

In this study significant proportion of health care provider was found to have psychological distress. Although, protecting the mental well-being of health care workers is a vital component of public health measures for preventing COVID-19 pandemic outbreak, still adequate psychological assistance for health care workers is lacking in Ethiopia. Due attention should be given to interventions needed to promote psychological well-being of health care workers during a COVID-19 era with particular attention to frontline healthcare workers who currently used substance and have depressive symptoms.

## Data Availability Statement

The raw data supporting the conclusions of this article will be made available by the authors, without undue reservation.

## Ethics Statement

The study was carried out after ethical clearance was obtained from the ethical review committee of the College of health science of Mettu University (RCS/012/2012) which conformed to the principles embodied in the Declaration of Helsinki. An approbation letter was obtained from the head of the department of psychiatry. Health care providers were told about the nature, purposes, benefits, and adverse effects of the study and were invited to participate. Confidentiality was ensured and all related questions, they raised were answered and all respondents provided written informed consent.

## Author Contributions

All authors were contributed to the inception of the study, organized the data collection process, equally contributed to data analysis, drafting or revising the article, gave final approval of the version to be published, and agree to be accountable for all aspects of the work.

## Conflict of Interest

The authors declare that the research was conducted in the absence of any commercial or financial relationships that could be construed as a potential conflict of interest.

## Abbreviations

CI: Confidence Interval
COVID 19 –: Corona Virus Disease 2019
DASS-21: Depression, Anxiety and Stress Scale 21 item
DSM-5: Diagnostic and Statistical Manual of mental disorders Fifth Edition
HCP: Health Care Provider
HW: Health Workers
IES-R: Impact of Event Scale
OR: Odds Ratio
PTSD: Post Traumatic Stress Disorder
SARS: Severe Acute Respiratory Syndrome
SPSS: Statistical Package for Social Science.
